# Practical Notes on Diagnosis and Treatment

**Published:** 1910-01-29

**Authors:** 


### Practical Notes on Diagnosis and Treatment.

**Rickets.**

Sweating about the head is of little or no diagnostic significance in rickets.—*Dr. Still.*

**Pericarditis in Children.**

Increasing cyanosis is one of the most important signs suggesting that the effused fluid has become purulent.—*Dr. D. Forsyth.*

**Thymol in Toothache.**

As a temporary measure in toothache arising from a hollow tooth a few grains of thymol will afford relief if placed in the cavity and covered with a little cotton wool.

**Agar-agar.**

Cases of chronic constipation have been much benefited by the admixture of agar-agar with a suitable diet. About a teaspoonful of the dry powder with two or three of the meals is an average dose.

**Rheumatic Nodules.**

The presence of these nodules generally means that there is some active heart disease, and although they may persist for months one should be cautious in allowing a child to resume an active life, while nodules are present. They are scarcely ever seen in a first attack.—*Dr. Abercrombie.*

**Morning Diarrhœa.**

This troublesome form of diarrhœa may be successfully combated by forbidding the patient to take any liquid at all after six o'clock in the evening. *Sir T. Lauder Brunton.*

**Knee-jerks in Pneumonia.**

In the majority of cases of lobar pneumonia the knee-jerks will be found to be absent during the height of the illness. In the diagnosis of atypical cases this observation is of some value; for, if the knee-jerks are brisk, the chances are that the mischief in the lungs is being caused by the tubercle bacillus, and not by the pneumococcus.—*Dr. C. Rolleston.*

**Canadian Hemp.**

The fluid extract of Apocynum, which is official in the United States pharmocopœia, is an excellent diuretic. It is valuable in cardiac dropsy, especially when there is ascites, and is also useful in cases of pleurisy with effusion. The dose should not exceed 10 minims. Some preparations are apt to produce excessive purgation owing to admixture of the fibre of the wood with the bark of the root, so the patient should be under daily observation.—*Dr. H. Green.*

